# Comparison of Dimensional Accuracy and Stability of 3D-Printed, Computer-Aided Design/Computer-Aided Manufacturing (CAD/CAM), and Conventional Polymethyl Methacrylate (PMMA) Denture Base Materials: An In Vitro Study

**DOI:** 10.7759/cureus.85128

**Published:** 2025-05-31

**Authors:** Priyanka Borse, Shashikala Jain, Navreet Bhasin, Ramanjeet K Grover, Balbir Singh, Pursenla Longkumer

**Affiliations:** 1 Department of Prosthodontics, Maharaja Ganga Singh Dental College and Research Centre, Sri Ganganagar, IND

**Keywords:** computer-aided design, denture bases, dimensional, polymethyl methacrylate, printing, stability, three-dimensional

## Abstract

Introduction: This in vitro experimental study aimed to evaluate and compare the dimensional accuracy and stability of polymethyl methacrylate (PMMA) denture base materials fabricated by three distinct techniques: conventional heat polymerization, computer-aided design/computer-aided manufacturing (CAD/CAM) milling, and three-dimensional (3D) printing. The objective of this study was to assess the influence of fabrication methods on the dimensional integrity of denture bases after processing and to simulate clinical conditions to determine which technique provides superior accuracy and minimal deformation over time.

Materials and methods: This in vitro experimental study was conducted at the Department of Prosthodontics, Maharaja Ganga Singh Dental College and Research Centre, Sri Ganganagar, Rajasthan, between May and December 2024. A master die (25 mm × 25 mm × 3 mm) was designed using AutoCAD 2024 (Autodesk Inc., USA) and milled using Plexiglass (Plazit Polygal India Pvt. Ltd., India). The Standard Tessellation Language (STL) file was used to cut a plexiglass (Plazit Polygal India Pvt. Ltd., India) template, which was then utilized for conventional compression molding. This process resulted in an actual master die, which was used to create molds for fabricating conventional PMMA specimens. The same STL file was used for fabricating CAD/CAM-milled and 3D-printed specimens, ensuring uniformity across all test specimens. The specimens were divided into three groups (n = 40 each). Group 1 used Triplex Hot PMMA (Ivoclar Vivadent AG, Liechtenstein) processed by compression molding and heat polymerization. Group 2 consisted of specimens CAD/CAM-milled from Ivotion Base discs (Ivoclar Vivadent AG, Liechtenstein) using a five-axis milling machine (Arum 5X-500, Doowon, South Korea). Group 3 included 3D-printed specimens fabricated from 3D Accuprint denture resin (D-Tech Dental Technologies, India) using a digital light processing (DLP) printer (Phrozen, Taiwan). All specimens were thermocycled 50 times between 5°C and 55°C (SD Mechatronik, Germany) and immersed in artificial saliva (Wet Mouth, ICPA Health Products Ltd., India) for seven days. Dimensional changes were evaluated using digital calipers (Mitutoyo, Japan) for 2D linear measurements and 3Shape extraoral scanning (3Shape A/S, Denmark) with Geomagic Control X (3D Systems, USA) for 3D superimposition.

Results: All the groups exhibited statistically significant reductions in length, width, and depth (p = 0.000). Intergroup comparisons showed no significant differences in the linear dimensions (p > 0.05). However, 3D superimposition showed significant differences (p = 0.000), with CAD/CAM demonstrating the least deviation (0.06 mm), followed by 3D printing (0.11 mm) and conventional printing (0.22 mm).

Conclusion: CAD/CAM milling showed superior dimensional accuracy and stability compared with 3D printing and conventional methods. Statistical analysis confirmed significant differences among the groups, with CAD/CAM-milled PMMA outperforming both alternatives. These findings support the use of CAD/CAM milling for precision-driven prosthodontics, with 3D-printed PMMA serving as a viable alternative for customization.

## Introduction

Polymethyl methacrylate (PMMA) has remained the cornerstone of denture base materials for decades owing to its favorable balance of esthetic appeal, biocompatibility, mechanical strength, and ease of manipulation [1]. Its ability to be precisely molded and polished, along with its translucency and color-matching properties, has made it the preferred choice for fabricating dentures that must be harmoniously integrated within the oral environment [1]. However, despite their longstanding clinical success, concerns regarding the dimensional accuracy and long-term stability of PMMA-based dentures continue to prompt innovation and comparisons among fabrication techniques [2].

Dimensional accuracy and stability are critical attributes for the functional and esthetic success of removable prostheses [3]. Accurate replication of anatomical structures ensures better adaptation of the denture to the underlying tissues, resulting in improved retention, support, and comfort [4]. Any deviation from the intended dimensions during fabrication can compromise prosthetic fit, leading to discomfort, poor masticatory efficiency, and the development of mucosal lesions [4]. Equally important is the dimensional stability of a material, which is its ability to resist distortion and maintain its shape under functional loads and over time [5]. Dimensional instability can lead to warping, loss of retention, and eventual failure of the prosthesis.

Conventional heat-polymerized PMMA is widely used because of its cost-effectiveness and clinical familiarity. This traditional technique involves a chemical reaction between a monomer and a polymer initiated by heat [2]. However, this process is susceptible to polymerization shrinkage and thermal contraction, which can lead to inaccuracies in the final denture base. Multiple studies have highlighted the limitations of this method, noting that dimensional changes during curing and cooling may compromise the fit and long-term adaptation of the prosthesis [6-8].

Digital technologies, such as computer-aided design and computer-aided manufacturing (CAD/CAM), have transformed the prosthetic dentistry landscape. CAD/CAM milling involves subtractive fabrication, in which prepolymerized PMMA blocks are milled into a shape. This method minimizes polymerization-related issues and has demonstrated higher precision and superior mechanical properties compared with conventionally fabricated dentures [9]. The uniformity of the prepolymerized PMMA blocks also results in lower porosity and increased flexural strength, contributing to enhanced durability and clinical performance [10].

Additive manufacturing techniques, particularly three-dimensional (3D) printing, have gained attention as versatile, efficient, and customizable options for denture fabrication [11]. Technologies such as stereolithography (SLA) and digital light processing (DLP) offer high-resolution layer-by-layer printing of complex geometries [11]. Although 3D printing presents promising advantages in terms of speed, customization, and reduced material waste, concerns have been raised regarding the consistency of the material properties and dimensional stability of printed dentures, especially under functional stress [12]. Layer-by-layer polymerization and post-curing processes may introduce variations that can impact the fit and long-term performance [12].

As digital workflows continue to evolve and become more accessible in clinical practice, there is a growing need to objectively assess and compare the outcomes of these fabrication techniques [11]. This in vitro study aimed to evaluate and compare the dimensional accuracy and stability of PMMA denture bases fabricated using three distinct methods: conventional heat polymerization, CAD/CAM milling, and 3D printing. By systematically analyzing the advantages and limitations of each technique, this study would provide clinicians with evidence-based insights to inform material and method selection, ultimately enhancing prosthetic outcomes and patient satisfaction. The null hypothesis posited for this study was that there would not be any statistically significant differences between the groups.

## Materials and methods

Study design

This in vitro experimental study was conducted at the Department of Prosthodontics, Maharaja Ganga Singh Dental College and Research Centre, Sri Ganganagar, Rajasthan, from May 2024 to December 2024. Because this in vitro study did not involve human participants or tissues, ethical approval was not required.

Sample size calculation

The sample size was calculated using the G*Power 3.1 software (Heinrich Heine University, Düsseldorf, Germany). The calculation was based on a significance level (α) of 0.05, power (1-β) of 0.80, and effect size derived from a pilot study. A one-way analysis of variance (ANOVA) test was used for intergroup comparisons, and the minimum required sample size was determined to be 34 per group. To account for potential errors or exclusions, the final sample size was increased to 40 per group.

Group allocation

The specimens were divided into three groups according to the type of denture base material and the fabrication technique. Group 1 included specimens made of conventional heat-polymerized PMMA resin (Triplex Hot; Ivoclar Vivadent AG, Liechtenstein, Germany). Group 2 consisted of CAD/CAM-milled PMMA specimens fabricated from prepolymerized discs (Ivotion Base, Ivoclar Vivadent AG, Liechtenstein). Group 3 included specimens fabricated using a 3D-printed PMMA resin (3D Accuprint Denture, D-Tech Dental Technologies, India).

Methodology

A standard master die with dimensions of 25 mm × 25 mm × 3 mm was digitally designed using AutoCAD 2024 (Autodesk Inc., San Rafael, California, USA), and a Standard Tessellation Language (STL) file of the design was generated. The STL file was used to cut a Plexiglass (Plazit Polygal India Pvt. Ltd., India) template, which was then utilized for conventional compression molding. This process resulted in an actual master die, which was used to create molds for fabricating conventional PMMA specimens. The same STL file was used for fabricating CAD/CAM-milled and 3D-printed specimens, ensuring uniformity across all test specimens.

In Group 1, the Plexiglass master die was coated with petroleum jelly and invested in a vacuum-mixed dental stone using a varsity flask. After dewaxing, two layers of die spacer were applied, and Triplex Hot PMMA resin was mixed according to the manufacturer's guidelines. The resin was packed under a pressure of 14.71 kN using a hydraulic press (Force, Hi-Force Ltd., UK) and polymerized in a thermostatically controlled water bath (3 Hot, Macrodent, India) following a short curing cycle of two hours at 74°C, followed by one hour at 100°C. After polymerization, the specimens were bench-cooled, deflasked, trimmed using tungsten carbide burs, and polished with rubber acrylic burs, a pumice slurry, and a rouge. Only one surface of each specimen was polished to simulate a tissue contact surface. The final dimensions were confirmed using a digital Vernier caliper (Mitutoyo Corporation, Japan). The specimens were then stored in distilled water for 48 hours before testing.

In Group 2, the STL file of the master die was imported into Exocad software (Align Technology, Arizona, USA). The specimens were then milled from votion-based PMMA discs using a five-axis milling machine (Arum 5X-500, Doowon, South Korea) under wet conditions. The specimens were finished using tungsten carbide burs and 400-grit silicon carbide paper for 10 seconds and then polished using the same protocol as in Group 1. The final dimensions were verified, and the specimens were stored in distilled water for 48 hours.

In Group 3, the STL file was transferred to a 3D printer (Phrozen, Taiwan) using DLP technology. 3D-printed PMMA resin was used, and the specimens were printed at a thickness of 50 µm. Prior to printing, the resin was manually agitated by shaking the resin bottle for three minutes to ensure uniform mixing, and the specimens were oriented at 45°C to the build platform. After printing with a UV projector (385 nm), the specimens were cleaned with isopropyl alcohol (Thermo Fisher Scientific India Pvt. Ltd., India) and post-cured for 15 minutes in a UV light-curing box (Phrozen, Taiwan). The 15-minute post-curing duration was selected based on manufacturer guidelines to ensure optimal polymerization, mechanical strength, and dimensional stability of the 3D-printed PMMA specimens. The support structures were removed, and the specimens were finished and polished according to a standard protocol. All the specimens were stored in distilled water for 48 hours prior to testing.

To simulate intraoral thermal fluctuations, all the specimens were subjected to thermocycling using a thermocycler (SD Mechatronik Thermocycler THE-1100; Feldkirchen-Westerham, Germany). Each specimen underwent 50 thermal cycles between 5°C and 55°C, with a dwell time of 30 seconds in each bath and a transfer time of 10 seconds between baths. Following thermocycling, the specimens were immersed in artificial saliva (Wet Mouth, ICPA Health Products Ltd., India) for seven days to replicate the oral conditions.

The dimensional changes were evaluated using two-dimensional (2D) linear measurements and 3D superimposition techniques. In the 2D method, three reference points were marked on each specimen to assess dimensional changes along the X-Y (width), X-Z (height), and Y-Z (length) axes. Pre- and post-treatment measurements were obtained using a high-precision digital caliper (Mitutoyo, Japan; accuracy, ±0.01 mm), and dimensional differences were calculated.

**Figure 1:**
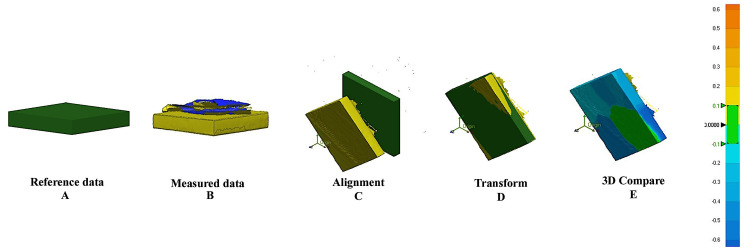
Superimposition and analysis of shrinkage material. 3D: three-dimensional. Image created by the authors.

For the 3D superimposition method, each specimen was scanned using a high-resolution extraoral scanner (3Shape A/S, Denmark; accuracy ±6 µm), and the resulting STL files were superimposed onto the original master STL file using the Geomagic Control X software (3D Systems, USA). The best-fit alignment method was applied, and dimensional deviations were visualized using color-coded heat maps. The green areas indicate minimal or no deviation, whereas the red and blue regions represent positive (expansion) and negative (shrinkage) deviations, respectively. Quantitative deviations were recorded in millimeters (Figure 1).

Statistical analysis

All collected data were statistically analyzed using the IBM SPSS Statistics for Windows, Version 26 (Released 2019; IBM Corp., Armonk, New York). Descriptive statistics, including the mean and standard deviation, were calculated. The Shapiro-Wilk test was used to assess the normality of the data distribution, and Levene's test was applied to evaluate the homogeneity of variance. For normally distributed data, a paired t-test for intragroup analysis and one-way ANOVA followed by Tukey's honest significant difference post-hoc test for intergroup comparisons were employed. Statistical significance was set at p < 0.05.

## Results

The null hypothesis was rejected as statistically significant dimensional changes (p = 0.0001) in all parameters across all groups post-intervention were noted. Conventional, CAD/CAM, and 3D-printed groups showed minimal but consistent reductions in length, depth, and width. While all groups demonstrated similar patterns of dimensional change, the CAD/CAM group showed slightly greater stability compared to the conventional and 3D-printed groups. These findings suggested that while all fabrication methods result in measurable dimensional changes, the CAD/CAM technique might offer marginally better dimensional stability than other alternatives (Table 1).

**Table 1:** Comparison of dimensional change after intervention using a paired t-test. *p-value < 0.05: significant; n = number of samples in the group. Data are presented in the form of mean ± standard deviation (SD). CAD/CAM: computer-aided design/computer-aided manufacturing, 3D: three-dimensional, PMMA: polymethyl methacrylate.

Group	Parameters	Initial	Final	t-stat	p-value
Conventional PMMA (n = 40)	Length (mm)	25.00 ± 0.000	24.97 ± 0.011	14.82	0.0001*
	Depth (mm)	25.00 ± 0.000	24.98 ± 0.008	10.46	0.0001*
	Width (mm)	3.00 ± 0.000	2.99 ± 0.004	12.82	0.0001*
CAD/CAM (n = 40)	Length (mm)	25.00 ± 0.000	24.98 ± 0.008	12.54	0.0001*
	Depth (mm)	25.00 ± 0.000	24.98 ± 0.010	11.60	0.0001*
	Width (mm)	3.00 ± 0.000	2.99 ± 0.003	10.71	0.0001*
3D-printed (n = 40)	Length (mm)	25.00 ± 0.000	24.97 ± 0.011	11.64	0.0001*
	Depth (mm)	25.00 ± 0.000	24.98 ± 0.015	7.14	0.0001*
	Width (mm)	3.00 ± 0.000	2.99 ± 0.006	6.45	0.0001*

Table 2 reveals no statistically significant differences in dimensional changes for length, depth, and width between conventional PMMA, CAD/CAM, and 3D-printed groups, with all showing similar mean changes. Overall, the results suggested comparable dimensional stability across fabrication methods for most parameters, though 3D printing exhibited slightly greater variability in width changes.

**Table 2:** Comparison of dimensional change between the study groups using one-way analysis of variance (ANOVA). p-value > 0.05: non-significant; n = number of samples in the group. Data are presented in the form of mean ± standard deviation (SD). CAD/CAM: computer-aided design/computer-aided manufacturing, 3D: three-dimensional, PMMA: polymethyl methacrylate, CI: confidence interval.

Parameters	Groups	Mean ± SD	95% CI for mean		F value	p-value
			Lower bound	Upper bound		
Length (mm)	Conventional PMMA (n = 40)	0.02 ± 0.01	0.02	0.02	0.95	0.388
	CAD/CAM (n = 40)	0.02 ± 0.01	0.02	0.03		
	3D-printed (n = 40)	0.02 ± 0.01	0.02	0.02		
Depth (mm)	Conventional PMMA (n = 40)	0.02 ± 0.01	0.01	0.02	0.21	0.821
	CAD/CAM (n = 40)	0.02 ± 0.01	0.01	0.02		
	3D-printed (n = 40)	0.02 ± 0.02	0.01	0.02		
Width (mm)	Conventional PMMA (n = 40)	0.01 ± 0.00	0.01	0.01	3.98	0.051
	CAD/CAM (n = 40)	0.01 ± 0.00	0.01	0.01		
	3D-printed (n = 40)	0.01 ± 0.00	0.00	0.01		

Table 3 results revealed highly significant differences in superimposition deviation among the three groups (p < 0.001). Analysis demonstrated that conventional PMMA showed the greatest deviation (0.224 ± 0.078 mm), followed by 3D-printed (0.111 ± 0.043 mm), with CAD/CAM exhibiting the least deviation (0.068 ± 0.025 mm). These findings suggest that CAD/CAM technology provides superior accuracy in dimensional maintenance compared to both conventional and 3D-printed methods, while 3D printing offers intermediate precision between conventional and CAD/CAM approaches.

**Table 3:** Comparison of superimposition deviation between groups. *p-value < 0.05: significant; n = number of samples in group. Data are presented in the form of mean ± standard deviation (SD). CAD/CAM: computer-aided design/computer-aided manufacturing, 3D: three-dimensional, PMMA: polymethyl methacrylate, CI: confidence interval.

Groups	n	Mean ± SD	95% CI for the Mean		F value	p-value
			Lower bound	Upper bound		
Conventional PMMA	40	0.224 ± 0.078	0.199	0.249	88.674	0.001*
CAD/CAM	40	0.068 ± 0.025	0.061	0.077		
3D-printed	40	0.111 ± 0.043	0.097	0.125		

Table 4 revealed statistically significant differences (p < 0.05) in superimposition deviation between all pairwise group comparisons. Conventional PMMA showed significantly greater deviation than both CAD/CAM and 3D-printed groups. CAD/CAM demonstrated superior accuracy with significantly lower deviation than both conventional PMMA and 3D-printed groups. The 3D-printed group exhibited intermediate performance, being significantly more accurate than conventional but less precise than CAD/CAM. These findings confirmed a clear hierarchy of dimensional accuracy: CAD/CAM > 3D-printed > conventional PMMA, with all differences being statistically and clinically significant.

**Table 4:** Pairwise comparison of groups using post-hoc Tukey's analysis. *p-value < 0.05: significant. The mean difference was calculated by subtracting the second group from the first group. CAD/CAM: computer-aided design/computer-aided manufacturing, 3D: three-dimensional, PMMA: polymethyl methacrylate, CI: confidence interval.

Pairwise Groups		Mean Difference	Standard Error	p-value	95% CI for the Mean	
					Lower bound	Upper bound
Conventional PMMA	CAD/CAM	0.155	0.012	0.000*	0.126	0.184
	3D-printed	0.112	0.012	0.000*	0.084	0.141
3D-printed	CAD/CAM	0.042	0.012	0.002*	0.013	0.071

## Discussion

The dimensional accuracy and stability of denture base materials are pivotal for ensuring optimal fit, retention, and patient comfort in prosthodontic applications. This study compared three fabrication methods, conventional heat-polymerized PMMA, CAD/CAM-milled PMMA, and 3D-printed PMMA, to evaluate their dimensional performance under simulated oral conditions, including thermal cycling and artificial saliva immersion.

The findings indicated that CAD/CAM-milled PMMA exhibited the highest dimensional stability, with the lowest mean surface deviation in 3D superimposition analysis and minimal changes in 2D linear measurements across the X, Y, and Z axes. Our findings were in accordance with previous studies [12,13]. These results align with the material's prepolymerized, high-density structure, which minimizes polymerization shrinkage and water absorption. The subtractive milling process ensures precise replication of digital designs, reducing porosity and residual monomer content, which are common issues in conventional PMMA [13]. A previous study by AlHelal et al. [14] had similarly reported that the industrial polymerization of CAD/CAM-milled PMMA enhanced its mechanical and dimensional properties, making it ideal for precision-driven applications such as complete dentures and implant-supported prostheses. The high accuracy of CAD/CAM-milled PMMA translates to better retention, fewer occlusal discrepancies, and a reduced need for clinical adjustments, offering significant advantages in clinical practice. The polymerization of CAD/CAM-milled PMMA under high pressure elevates its density while simultaneously decreasing porosity, thereby enhancing both flexural strength and fracture resistance [13]. According to Chuchulska et al. [15], CAD/CAM-milled PMMA exhibited superior flexural strength and impact resistance in comparison to traditional PMMA, rendering it more resilient for prolonged utilization. The fully polymerized CAD/CAM-milled PMMA, prior to the milling process, exhibits a markedly reduced residual monomer concentration, thereby enhancing its biocompatibility. The homogenous architecture attained through industrial manufacturing results in a more refined surface finish, thereby minimizing the necessity for extensive polishing [16].

However, in a study by Srinivasan et al. [17], it was found that the trueness of removable complete dentures fabricated with conventional techniques was similar to that of those fabricated with CAD/CAM. Notwithstanding these benefits, CAD/CAM-milled PMMA is not devoid of certain drawbacks. Its elevated cost can be attributed to the industrial processing involved and the necessity for specialized technological apparatus. Zupancic Cepic et al. [18] observed that while CAD/CAM-milled PMMA is characterized by an extended service life, its increased expense could pose challenges regarding accessibility for a subset of patients. Furthermore, the process of repair is not particularly straightforward; fractured restorations frequently necessitate comprehensive remanufacturing as opposed to uncomplicated chairside repairs. Smaller dental laboratories may encounter obstacles owing to the requirement for advanced milling machinery and sophisticated software [19].

In contrast, conventional heat-polymerized PMMA demonstrated the highest dimensional deviations, with the highest mean surface deviation and mean dimensional changes in the X, Y, and Z axes. This finding was supported by previous studies [9,10]. These findings reflect the material's susceptibility to polymerization shrinkage, thermal expansion, and water absorption, which are exacerbated by inconsistencies in manual processing [1,2]. The free-radical polymerization process often results in incomplete monomer conversion, leading to residual stresses and porosities that compromise dimensional stability [2]. Additionally, prolonged exposure to artificial saliva and thermal cycling induced significant expansion, consistent with findings by Figuerôa et al. [20], who noted that heat-polymerized PMMA undergoes measurable dimensional changes under hydration. These characteristics make conventional PMMA less reliable for applications requiring long-term precision, often necessitating frequent adjustments to maintain fit and comfort. Despite its affordability and widespread use, the material's limitations underscore the need for advanced fabrication techniques to meet modern prosthodontic demands.

3D-printed PMMA showed intermediate performance. While it outperformed conventional PMMA, it exhibited slightly higher deviations than CAD/CAM-milled PMMA, likely due to the layer-by-layer additive manufacturing process [12]. Gad et al. [21] emphasized that materials produced through 3D printing necessitate subsequent processing modifications to enhance their compatibility and mechanical characteristics. Furthermore, Kalberer et al. [12] conducted an assessment of the precision of dental models fabricated through CAD/CAM milling and 3D printing techniques, revealing that the milling process yielded superior accuracy, whereas the 3D printing process exhibited minor inaccuracies attributable to errors in layer deposition. Differential polymerization shrinkage between layers, combined with the resin's higher affinity for water absorption, may contribute to minor distortions [21]. However, the deviations remained within clinically acceptable limits, suggesting that 3D-printed PMMA is a viable alternative, particularly for applications prioritizing customization and material efficiency. The additive manufacturing process minimizes waste compared to CAD/CAM milling, which discards a significant amount of material during subtractive processing [22]. Previous studies have highlighted the sustainability benefits of 3D printing, noting material savings of up to 60%, which could reduce production costs and enhance accessibility [22]. Furthermore, 3D printing's ability to produce complex, patient-specific geometries offers unparalleled design flexibility, making it suitable for intricate prosthetic designs that are challenging to achieve with milling or conventional methods.

The clinical implications of these results are profound. The superior stability of a CAD/CAM-milled PMMA ensures predictable fit and minimal post-processing, reducing chairside adjustments and enhancing patient satisfaction. Its high precision is particularly advantageous for complex restorations, such as implant-supported prostheses, where even minor discrepancies can compromise functionality. 3D-printed PMMA, while slightly less stable, offers a compelling alternative for practices embracing digital workflows. Its rapid fabrication, customization capabilities, and material efficiency make it suitable for same-day restorations and resource-conscious settings. Conventional PMMA, despite its cost-effectiveness, may lead to suboptimal outcomes in precision-driven cases, as its dimensional instability can cause poor retention, occlusal imbalances, and soft tissue irritation. These drawbacks highlight the need to transition toward digital fabrication methods to meet evolving clinical standards.

The study's methodology, including thermal cycling and artificial saliva immersion, effectively simulated intraoral conditions, providing a robust framework for evaluating material performance. Thermal cycling, which alternated between 5°C and 55°C for 50 cycles, induced expansion and contraction stresses, revealing the materials' susceptibility to temperature fluctuations. Artificial saliva immersion mimicked moisture absorption and chemical interactions, critical factors influencing long-term stability. While these conditions accelerate aging, they may not fully replicate the mechanical stresses of mastication or patient-specific factors. This limitation underscores the need for complementary in vivo studies to validate the findings under real-world conditions.

Several limitations must be acknowledged. The in vitro design, while controlled, did not account for masticatory forces, saliva dynamics, or patient variability, which could influence dimensional stability. The short-term evaluation period limited insights into long-term performance, particularly for 3D-printed PMMA, where clinical data remained scarce. Additionally, the study focused on DLP for 3D printing, leaving other technologies, such as fused deposition modeling (FDM), unexplored. Future research should compare these systems to identify the most effective 3D printing approach. Incorporating advanced measurement techniques, such as micro-computed tomography or volumetric scanning, could also provide deeper insights into internal defects and volumetric changes.

## Conclusions

This study compared the dimensional accuracy and stability of conventional heat-polymerized, CAD/CAM-milled, and 3D-printed PMMA denture base materials under simulated oral conditions. CAD/CAM-milled PMMA demonstrated the highest dimensional stability, followed by 3D-printed PMMA, while conventional PMMA exhibited the greatest deviations. Statistical analysis confirmed significant differences among the groups, with CAD/CAM-milled PMMA outperforming both alternatives. These findings support the use of CAD/CAM milling for precision-driven prosthodontics, with 3D-printed PMMA serving as a viable alternative for customization. Conventional PMMA is less reliable due to shrinkage and water absorption. Future research should optimize 3D printing and evaluate long-term clinical outcomes.
